# 983. Use of plasma-based microbial cell-free DNA (mcfDNA) sequencing for surveillance of infection in the 1st month after lung transplant (LT): a prospective, observational, pilot study

**DOI:** 10.1093/ofid/ofad500.038

**Published:** 2023-11-27

**Authors:** Ghady Haidar, Kelly Friday, Christopher Musgrove, Xiaohong Wang, Angela Pisarra, Kailey Hughes Kramer, Kentaro Noda, Naomi Ryssel, Andrew Craig, Timothy A Blauwkamp, Matthew Morrell, Chadi Hage, Mark Snyder, Pablo Sanchez, Alison Morris, Georgios Kitsios

**Affiliations:** University of Pittsburgh School of Medicine, Pittsburg, PA; University of Pittsburgh, Pittsburgh, Pennsylvania; University of Pittsburgh, Pittsburgh, Pennsylvania; University of Pittsburgh, Pittsburgh, Pennsylvania; University of Pittsburgh, Pittsburgh, Pennsylvania; Division of Infectious Diseases, Department of Medicine, University of Pittsburgh School of Medicine, Pittsburgh, Pennsylvania; University of Pittsburgh, Pittsburgh, Pennsylvania; UPMC, Glenshaw, Pennsylvania; University of Pittsburgh, Pittsburgh, Pennsylvania; Karius Inc, Redwood City, California; University of Utah, Salt lake city, Utah; University of Pittsburgh, Pittsburgh, Pennsylvania; University of Pittsburgh, Pittsburgh, Pennsylvania; University of Pittsburgh, Pittsburgh, Pennsylvania; University of Pittsburgh Medical Center, Pittsburgh, PA; University of Pittsburgh Medical Center, Pittsburgh, PA

## Abstract

**Background:**

Infections after LT are common, but standard of care testing (SOCT) often fails to identify a cause. We prospectively compared longitudinal mcfDNA sequencing vs SOCT to determine the utility of mcfDNA sequencing for infection surveillance after LT.

**Methods:**

Single center prospective observational study of new LT patients (pts). We collected plasma on days 0, 1, 2, 3, 7, 14, and 30 post LT for mcfDNA sequencing; results were not available clinically. We reviewed outcomes and SOCT through 6 months (mo).

**Results:**

We enrolled 30 LT pts (4/2021-12/2022, Table 1). 93.3% (28/30) received broad-spectrum antibiotics, although only 40.0% (12/30) had SOCT yielding pathogenic bacteria. 43.33% (13/30) had unexplained leukocytosis. Table 2 shows serial mcfDNA sequencing results, clinical course, and adjudication of mcfDNA sequencing. 96% (29/30) had at least 1 positive mcfDNA sequencing test, including 68.75% (11/16) with presumed infection or unexplained leukocytosis and negative SOCT, but diversity of organisms on mcfDNA sequencing, e.g., *Candida*, oral or gastrointestinal commensals, *Enterococci*, or obligate pathogens (*Enterobacter, Pseudomonas, Mycoplasma, Ureaplasma*, others); most had polymicrobial mcfDNA sequencing results. In 36.67% (11/30), mcfDNA sequencing detected pathogens of unclear significance (potential false positive, FP). In 8 pts with positive SOCT, mcfDNA sequencing did not detect at least 1 pathogen found on SOCT. In 13% (4/30), mcfDNA sequencing detected a pathogen before it was detected by SOCT (n=1 each): VRE surgical site infection (SSI), invasive candidiasis, *Enterobacter* pneumonia, and *M. hominis* SSI (a known donor-derived syndrome) with HHV-8/Kaposi Sarcoma [presumed donor-derived due to donor risk factors, diagnosed on autopsy, pt 19].

Table 1.

**Figure:**
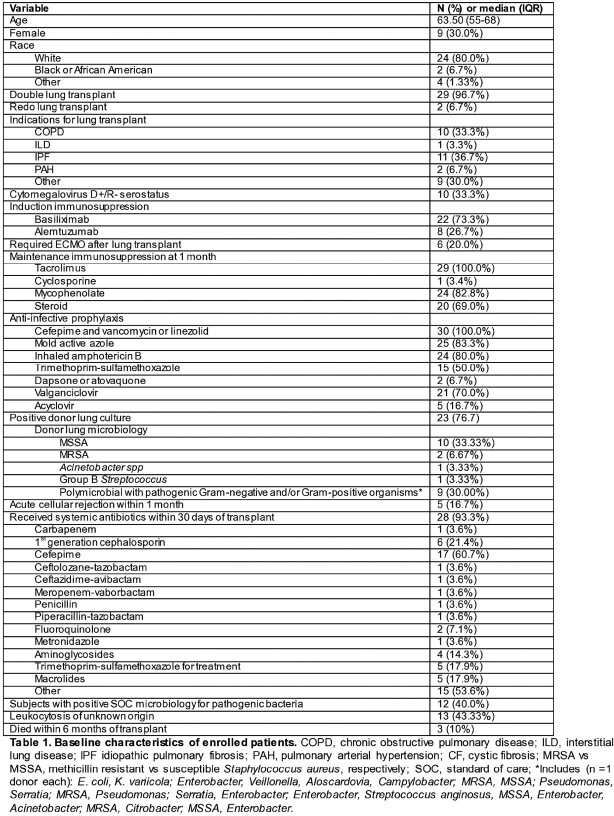

Table 2 legend.

**Figure:**
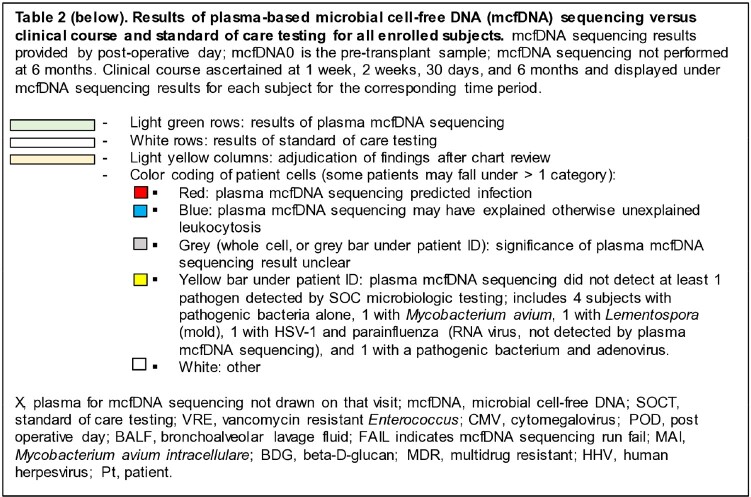

Table 2.

**Figure:**
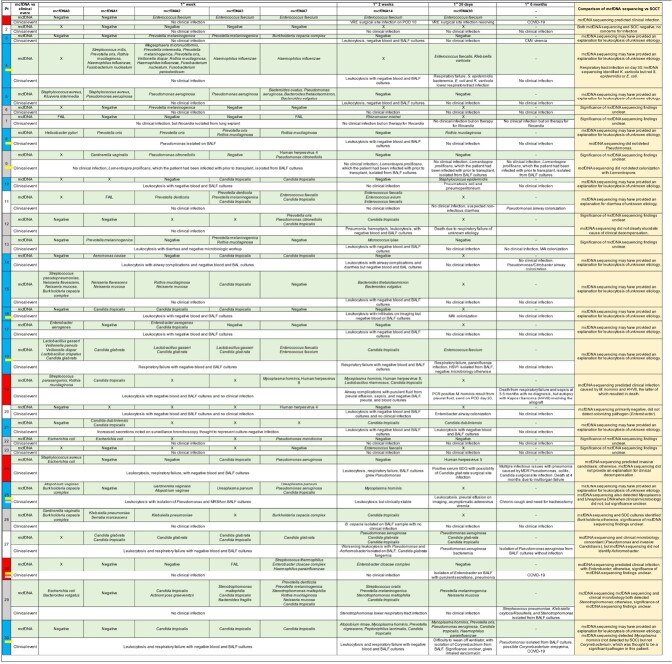

**Conclusion:**

Plasma mcfDNA sequencing revealed potential occult explanations for unexplained leukocytosis and infectious syndromes with negative SOCT early post LT. mcfDNA sequencing also detected infection earlier than SOCT in 4 pts but did not detect known pathogens from 8 pts; > 1/3 had presumed FP mcfDNA sequencing results. Larger studies with longer follow-up are needed to determine the utility of mcfDNA sequencing in diagnosing infection, optimizing antibiotic use, and monitoring donor-derived infection post LT.

**Disclosures:**

**Ghady Haidar, MD**, Allovir: Grant/Research Support|AstraZeneca: Advisor/Consultant|AstraZeneca: Grant/Research Support|Karius: Advisor/Consultant|Karius: Grant/Research Support|NIH: Grant/Research Support **Timothy A. Blauwkamp, PhD**, Karius: Board Member|Karius: Ownership Interest **Georgios Kitsios, MD, PhD**, Karius, Inc: Grant/Research Support

